# P-1375. Evaluation of Comprehensive STI Screening in the Emergency Department Setting

**DOI:** 10.1093/ofid/ofae631.1551

**Published:** 2025-01-29

**Authors:** Braden Sciarra, Katherine Frasca, Brian T Montague

**Affiliations:** University of Colorado, Denver, Colorado; University of Colorado School of Medicine, Aurora, Colorado; University of Colorado School of Medicine, Aurora, Colorado

## Abstract

**Background:**

Sexually transmitted infections (STI) are a common reason for emergency department (ED) evaluation. Patients diagnosed with or suspected to have an STI are also at risk for other STIs, and should be screened in accordance with Center for Disease Control (CDC) recommendations.

**Figure 1:**
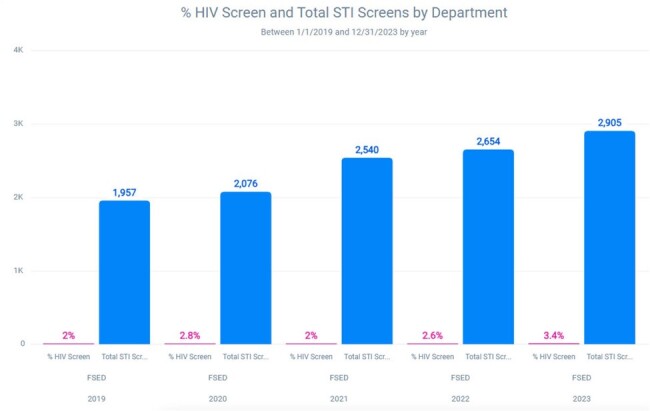
Percentage of HIV screenings among ED patients tested for STIs

**Methods:**

This study is a retrospective analysis of comprehensive STI screening conducted at eleven freestanding emergency departments associated with an academic referral center. Individuals tested for gonorrhea, chlamydia, and syphilis between 1/1/2019 and 12/31/2023 in the emergency department were evaluated for the completion of appropriate comprehensive STI screening, defined as the completion of HIV co-testing during the same ED encounter. Additional evaluation of HIV testing was also completed in higher risk individuals that tested positive for gonorrhea and/or chlamydia.

**Figure 2:**
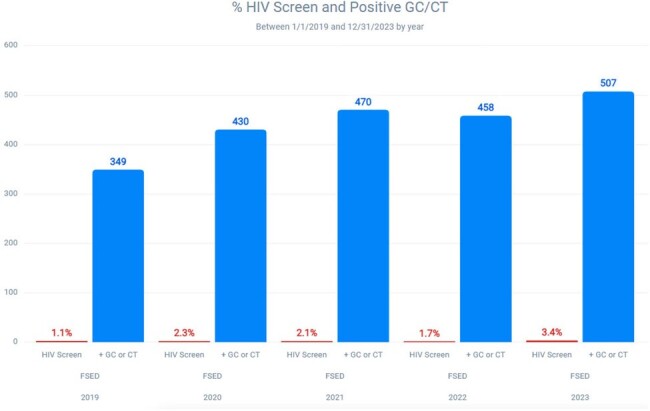
Percentage of HIV screenings among ED patients testing positive for gonorrhea or chlamydia

**Results:**

The analysis showed that comprehensive STI screening in the emergency department was completed in only 2.9% of the 10,895 patients that were screened for gonorrhea, chlamydia, or syphilis. The rate of comprehensive STI screening in the emergency department was 2.6% among the 2,082 patients that tested positive for gonorrhea and/or chlamydia.

**Conclusion:**

The CDC recommends HIV screening for all persons that present for evaluation of possible sexually transmitted infections. Our analysis of individuals undergoing STI screening in the ED demonstrates a significant gap in HIV screening, including amongst higher risk individuals that have tested positive for gonorrhea and/or chlamydia. Based on these results, a comprehensive STI screening educational tool was developed within the electronic medical record to increase comprehensive STI screening. Further analysis of these interventions will be studied in the future.

**Disclosures:**

**Brian T. Montague, DO MS MPH**, Regeneron: Grant/Research Support

